# 2-Amino-3-carb­oxy­pyridinium nitrate

**DOI:** 10.1107/S1600536811027978

**Published:** 2011-07-16

**Authors:** Fadila Berrah, Sofiane Bouacida, Thierry Roisnel

**Affiliations:** a Laboratoire de Chimie Appliquée et Technologie des Matériaux LCATM, Université Larbi Ben M’hidi, 04000 Oum El Bouaghi, Algeria; b Unité de Recherche de Chimie de l’Environnement et Moléculaire Structurale, CHEMS, Faculté des Sciences Exactes, Université Mentouri Constantine 25000, Algeria.; c Centre de Difractométrie X, UMR 6226 CNRS Unité Sciences Chimiques de Rennes, Université de Rennes I, 263 Avenue du Général Leclerc, 35042 Rennes, France

## Abstract

In the crystal structure of the title compound, C_6_H_7_N_2_O_2_^+^·NO_3_^−^, the cations are linked *via* C—H⋯O hydrogen bonds, forming infinite chains running along the *b* axis. These chains are further linked through N—H⋯O, O—H⋯O and C—H⋯O hydrogen bonds to the nitrate anions, forming well-separated infinite planar layers parallel to (001).

## Related literature

For hybrid compounds based on nicotinic acid, see: Athimoolam et al. (2005 ▶); Athimoolam & Rajaram (2005*a* ▶,*b* ▶); Chen (2009 ▶); Slouf (2001 ▶); Ye et al. (2010 ▶). For hybrid compounds based on amino-nicotinic acid derivatives, see: Akriche & Rzaigui (2007 ▶); Berrah et al. (2011*a* ▶); Giantsidis & Turnbull (2000 ▶). For related nitrate compounds, see: Berrah et al. (2011*b* ▶); Jebas et al. (2006 ▶).
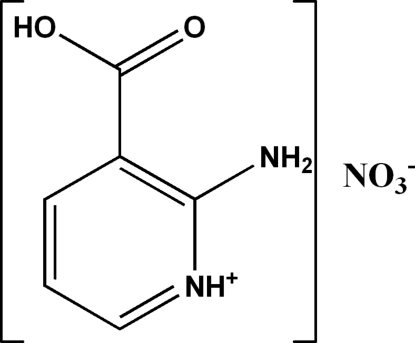


## Experimental

### 

#### Crystal data

C_6_H_7_N_2_O_2_^+^·NO_3_^−^
*M* *_r_* = 201.15
Tetragonal, 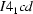

*a* = 16.122 (2) Å
*c* = 12.446 (3) Å
*V* = 3235.0 (11) Å^3^
*Z* = 16
Mo *K*α radiation
μ = 0.15 mm^−1^
*T* = 150 K
0.39 × 0.07 × 0.05 mm

#### Data collection

Bruker APEXII diffractometer
Absorption correction: multi-scan (*SADABS*; Sheldrick, 2002 ▶) *T*_min_ = 0.476, *T*_max_ = 0.993
5438 measured reflections
1509 independent reflections
921 reflections with *I* > 2σ(*I*)
*R*_int_ = 0.112

#### Refinement

*R*[*F* ^2^ > 2σ(*F* ^2^)] = 0.059
*wR*(*F* ^2^) = 0.158
*S* = 0.97
1509 reflections
128 parameters
1 restraint
H-atom parameters constrained
Δρ_max_ = 0.41 e Å^−3^
Δρ_min_ = −0.28 e Å^−3^

### 

Data collection: *APEX2* (Bruker, 2006 ▶); cell refinement: *SAINT* (Bruker, 2006 ▶); data reduction: *SAINT*; program(s) used to solve structure: *SIR2002* (Burla *et al.*, 2005 ▶); program(s) used to refine structure: *SHELXL97* (Sheldrick, 2008 ▶); molecular graphics: *ORTEP-3 for Windows* (Farrugia, 1997 ▶) and *DIAMOND* (Brandenburg & Berndt, 2001 ▶); software used to prepare material for publication: *WinGX* (Farrugia, 1999 ▶).

## Supplementary Material

Crystal structure: contains datablock(s) global, I. DOI: 10.1107/S1600536811027978/lx2193sup1.cif

Structure factors: contains datablock(s) I. DOI: 10.1107/S1600536811027978/lx2193Isup2.hkl

Supplementary material file. DOI: 10.1107/S1600536811027978/lx2193Isup3.cml

Additional supplementary materials:  crystallographic information; 3D view; checkCIF report

## Figures and Tables

**Table 1:** Hydrogen-bond geometry (Å, °)

*D*—H⋯*A*	*D*—H	H⋯*A*	*D*⋯*A*	*D*—H⋯*A*
N2—H2⋯O3^i^	0.86	1.97	2.803 (4)	162
N3—H3*A*⋯O2^i^	0.86	2.18	3.017 (4)	165
N3—H3*A*⋯O2^ii^	0.86	2.43	2.967 (4)	121
N3—H3*B*⋯O4	0.86	2.10	2.716 (5)	128
O5—H51⋯O3^iii^	0.82	1.85	2.670 (4)	180
C4—H4⋯O1^iv^	0.93	2.42	3.197 (6)	141
C5—H5⋯O4^v^	0.93	2.30	3.216 (6)	167

Symmetry codes: (i) 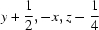; (ii) 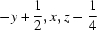; (iii) 
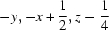
; (iv) 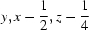; (v) 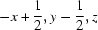.

**Fig. 1.**
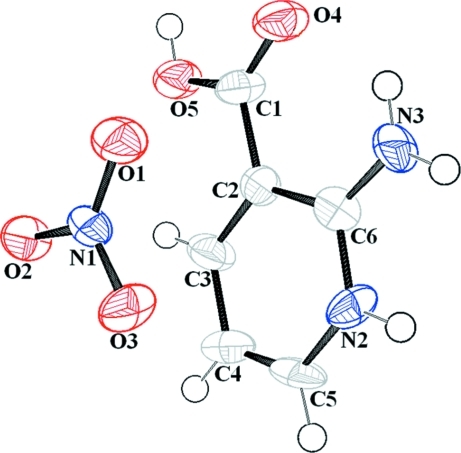
The structure of the title compound with the atomic labelling scheme. Displacement are drawn at the 50% probability level.

**Fig. 2.**
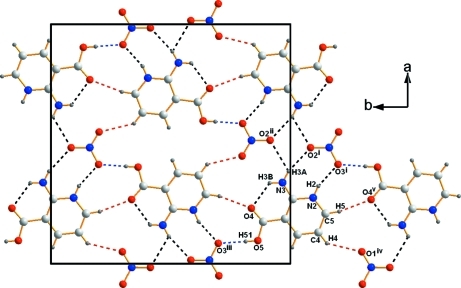
A part of crystal packing showing cationic infinite chains linked to nitrate anions via [N–H···O, O–H···O and C–H···O] hydrogen contacts. Hydrogen bonds are shown as dashed lines. [Symmetry codes: (i) y + 1/2, -x, z - 1/4; (ii) -y + 1/2, x, z - 1/4; (iii) -y, -x + 1/2, z - 1/4; (iv) y, x - 1/2, z - 1/4; (v) -x + 1/2, y - 1/2, z.]

**Fig. 3.**
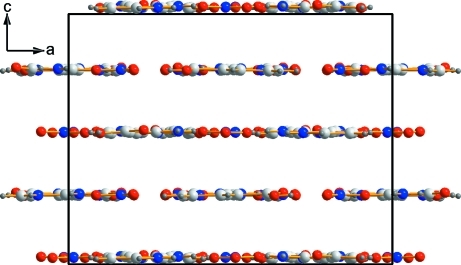
Layered packing of the structure viewed down the b axis.

## supplementary crystallographic information

### Comment

Crystal structures of hybrid compounds based on nicotinic acid or its amino derivatives and inorganic acids have been reported (Athimoolam *et al.*, 2005; Athimoolam & Rajaram, 2005**a*, b*; Chen, 2009; Giantsidis & Turnbull, 2000; Jebas *et al.*, 2006; Slouf, 2001; Ye *et al.*, 2010) showing interesting structural diversity governed mainly by hydrogen bonds. Anion substitution seems to have an important influence on hydrogen bound patterns. In attempt to elucidate this influence and as part of our search for new hybrid compounds based on protonated N-hyterocycle, we report in this paper the new structure of 2-aminonicotinium nitrate; its homologues obtained with phosphate and sulfate anions have been described previously (Akriche & Rzaigui, 2007; Berrah *et al.*, 2011*a*).

The asymmetric unit of the title compound (Fig. 1) contains one cation and one anion with geometry similar to that observed in similar compounds (Akriche & Rzaigui, 2007; Berrah *et al.*, 2011**a*, b*; Jebas *et al.*, 2006). However in this structure, cations do not form dimers *via* N–H···O hydrogen bonds as observed in the structures obtained with phosphate and sulfate anions but they are linked to each other *via* C–H···O hydrogen bonds to form infinite chains running along the *b* axis (Table 1 & Fig. 2,). These chains are further linked, through N–H···O, O–H···O and C–H···O hydrogen contacts, to nitrate anions to form well separated infinite planar layers parallel to (001) (Fig. 3). A such two-dimensional network have been already observed in compounds including nicotinium entities (Giantsidis & Turnbull, 2000; Slouf, 2001; Ye *et al.*, 2010).

### Experimental

The title compound was synthesized by reacting 3-amino-pyridine-2-carboxylic acid (0.138 mg, 1 mmol) with nitriic acid (1 mmol)in a solution of equal volume of H_2_O and CH_3_OH. Slow evaporation leads to well crystallized colourless needles.

### Refinement

All the Friedel pairs were merged. All non-H atoms were refined with anisotropic atomic displacement parameters. The remaining H atoms were localized on Fourier maps but introduced in calculated positions and treated as riding on their parent atoms (C, N or O) with C–H = 0.93 Å, O–H = 0.82 Å and N–H = 0.86 Å with *U*_iso_(H) = 1.2 *U*_eq_(C or N) and *U*_iso_(H = 1.5 *U*_eq_(O).

### Crystal data

**Table:**

C_6_H_7_N_2_O_2_^+^·NO_3_^−^	*D*_x_ = 1.652 Mg m^−^^3^
*M**_r_* = 201.15	Mo *K*α radiation, λ = 0.71073 Å
Tetragonal, *I*4_1_*c**d*	Cell parameters from 491 reflections
Hall symbol: I 4bw -2c	θ = 3.3–20.3°
*a* = 16.122 (2) Å	µ = 0.15 mm^−^^1^
*c* = 12.446 (3) Å	*T* = 150 K
*V* = 3235.0 (11) Å^3^	Needle, colourless
*Z* = 16	0.39 × 0.07 × 0.05 mm
*F*(000) = 1664	

### Data collection

**Table:**

Bruker APEXII diffractometer	921 reflections with *I* > 2σ(*I*)
graphite	*R*_int_ = 0.112
CCD rotation images, thin slices scans	θ_max_ = 27.5°, θ_min_ = 3.3°
Absorption correction: multi-scan (*SADABS*; Sheldrick, 2002)	*h* = −16→20
*T*_min_ = 0.476, *T*_max_ = 0.993	*k* = −13→20
5438 measured reflections	*l* = −16→10
1509 independent reflections	

### Refinement

**Table:**

Refinement on *F*^2^	Primary atom site location: structure-invariant direct methods
Least-squares matrix: full	Secondary atom site location: difference Fourier map
*R*[*F*^2^ > 2σ(*F*^2^)] = 0.059	Hydrogen site location: difference Fourier map
*wR*(*F*^2^) = 0.158	H-atom parameters constrained
*S* = 0.97	*w* = 1/[σ^2^(*F*_o_^2^) + (0.076*P*)^2^] where *P* = (*F*_o_^2^ + 2*F*_c_^2^)/3
1509 reflections	(Δ/σ)_max_ < 0.001
128 parameters	Δρ_max_ = 0.41 e Å^−^^3^
1 restraint	Δρ_min_ = −0.28 e Å^−^^3^

### Special details

**Table:**

Geometry. All e.s.d.'s (except the e.s.d. in the dihedral angle between two l.s. planes) are estimated using the full covariance matrix. The cell e.s.d.'s are taken into account individually in the estimation of e.s.d.'s in distances, angles and torsion angles; correlations between e.s.d.'s in cell parameters are only used when they are defined by crystal symmetry. An approximate (isotropic) treatment of cell e.s.d.'s is used for estimating e.s.d.'s involving l.s. planes.
Refinement. Refinement of *F*^2^ against ALL reflections. The weighted *R*-factor *wR* and goodness of fit *S* are based on *F*^2^, conventional *R*-factors *R* are based on *F*, with *F* set to zero for negative *F*^2^. The threshold expression of *F*^2^ > σ(*F*^2^) is used only for calculating *R*-factors(gt) *etc*. and is not relevant to the choice of reflections for refinement. *R*-factors based on *F*^2^ are statistically about twice as large as those based on *F*, and *R*- factors based on ALL data will be even larger.

### Fractional atomic coordinates and isotropic or equivalent isotropic displacement parameters (Å^2^)

**Table:**

	*x*	*y*	*z*	*U*_iso_*/*U*_eq_	
C1	0.1754 (3)	0.1137 (3)	0.0309 (6)	0.0288 (11)	
C2	0.1875 (3)	0.0223 (2)	0.0276 (6)	0.0229 (10)	
C3	0.1218 (3)	−0.0300 (3)	0.0259 (6)	0.0295 (12)	
H3	0.0686	−0.0078	0.0264	0.035*	
C4	0.1311 (3)	−0.1174 (3)	0.0233 (7)	0.0309 (11)	
H4	0.0854	−0.1526	0.0215	0.037*	
C5	0.2092 (3)	−0.1469 (3)	0.0235 (7)	0.0302 (12)	
H5	0.2179	−0.2039	0.0221	0.036*	
C6	0.2702 (2)	−0.0099 (3)	0.0279 (10)	0.0234 (10)	
N1	0.1623 (2)	−0.0149 (2)	0.2777 (8)	0.0247 (8)	
N2	0.2755 (2)	−0.0950 (2)	0.0256 (5)	0.0269 (9)	
H2	0.3242	−0.1168	0.0256	0.032*	
N3	0.3388 (2)	0.0327 (2)	0.0312 (5)	0.0308 (10)	
H3A	0.3858	0.0075	0.032	0.037*	
H3B	0.3371	0.086	0.0326	0.037*	
O1	0.20156 (19)	0.05016 (19)	0.2755 (4)	0.0313 (8)	
O2	0.08451 (16)	−0.01536 (18)	0.2757 (5)	0.0293 (8)	
O3	0.19907 (18)	−0.08530 (18)	0.2798 (5)	0.0309 (8)	
O5	0.09671 (19)	0.13575 (18)	0.0242 (4)	0.0342 (9)	
H51	0.0932	0.1865	0.0259	0.051*	
O4	0.2318 (2)	0.1628 (2)	0.0360 (4)	0.0355 (10)	

### Atomic displacement parameters (Å^2^)

**Table:**

	*U*^11^	*U*^22^	*U*^33^	*U*^12^	*U*^13^	*U*^23^
C1	0.035 (3)	0.017 (2)	0.035 (3)	−0.002 (2)	−0.001 (4)	−0.004 (3)
C2	0.020 (2)	0.021 (3)	0.028 (2)	0.0022 (16)	0.001 (3)	0.002 (4)
C3	0.025 (3)	0.021 (2)	0.042 (3)	−0.0068 (19)	0.000 (4)	0.003 (3)
C4	0.042 (3)	0.016 (3)	0.035 (3)	−0.001 (2)	−0.003 (5)	0.000 (3)
C5	0.040 (3)	0.014 (2)	0.036 (3)	−0.014 (2)	−0.004 (4)	−0.001 (4)
C6	0.022 (2)	0.026 (3)	0.022 (2)	−0.0025 (18)	−0.006 (5)	0.003 (3)
N1	0.0198 (18)	0.020 (2)	0.0341 (18)	−0.0058 (16)	0.006 (4)	−0.001 (4)
N2	0.024 (2)	0.022 (2)	0.035 (2)	0.0103 (15)	−0.005 (3)	−0.002 (3)
N3	0.0137 (19)	0.029 (2)	0.050 (2)	−0.0035 (15)	0.003 (3)	0.002 (3)
O1	0.0253 (18)	0.0256 (17)	0.043 (2)	−0.0062 (15)	0.000 (3)	−0.006 (3)
O2	0.0124 (15)	0.0231 (18)	0.052 (2)	0.0030 (13)	0.004 (3)	0.004 (3)
O3	0.0130 (16)	0.0233 (17)	0.056 (2)	0.0049 (13)	−0.002 (3)	−0.007 (3)
O5	0.0233 (17)	0.0205 (19)	0.059 (2)	0.0042 (14)	0.004 (3)	0.000 (3)
O4	0.0253 (19)	0.0212 (18)	0.060 (3)	0.0027 (15)	−0.003 (3)	−0.005 (3)

### Geometric parameters (Å, °)

**Table:**

C1—O4	1.207 (6)	C5—H5	0.93
C1—O5	1.319 (6)	C6—N3	1.303 (5)
C1—C2	1.487 (6)	C6—N2	1.375 (6)
C2—C3	1.354 (6)	N1—O1	1.225 (4)
C2—C6	1.431 (5)	N1—O2	1.255 (4)
C3—C4	1.417 (6)	N1—O3	1.281 (4)
C3—H3	0.93	N2—H2	0.86
C4—C5	1.347 (8)	N3—H3A	0.86
C4—H4	0.93	N3—H3B	0.86
C5—N2	1.359 (6)	O5—H51	0.82
			
O4—C1—O5	123.4 (4)	N2—C5—H5	119.4
O4—C1—C2	123.5 (4)	N3—C6—N2	118.2 (4)
O5—C1—C2	113.0 (4)	N3—C6—C2	126.9 (5)
C3—C2—C6	120.2 (4)	N2—C6—C2	114.8 (4)
C3—C2—C1	121.0 (4)	O1—N1—O2	121.4 (4)
C6—C2—C1	118.8 (4)	O1—N1—O3	121.4 (3)
C2—C3—C4	122.5 (4)	O2—N1—O3	117.2 (3)
C2—C3—H3	118.8	C5—N2—C6	124.5 (4)
C4—C3—H3	118.8	C5—N2—H2	117.8
C5—C4—C3	116.8 (4)	C6—N2—H2	117.8
C5—C4—H4	121.6	C6—N3—H3A	120
C3—C4—H4	121.6	C6—N3—H3B	120
C4—C5—N2	121.2 (4)	H3A—N3—H3B	120
C4—C5—H5	119.4	C1—O5—H51	109.5
			
O4—C1—C2—C3	177.5 (7)	C3—C2—C6—N3	−178.8 (9)
O5—C1—C2—C3	−4.4 (11)	C1—C2—C6—N3	0.4 (17)
O4—C1—C2—C6	−1.6 (13)	C3—C2—C6—N2	0.3 (14)
O5—C1—C2—C6	176.4 (9)	C1—C2—C6—N2	179.5 (7)
C6—C2—C3—C4	−0.6 (12)	C4—C5—N2—C6	0.0 (14)
C1—C2—C3—C4	−179.7 (7)	N3—C6—N2—C5	179.1 (8)
C2—C3—C4—C5	0.5 (11)	C2—C6—N2—C5	−0.1 (15)
C3—C4—C5—N2	−0.3 (12)		

### Hydrogen-bond geometry (Å, °)

**Table:**

*D*—H···*A*	*D*—H	H···*A*	*D*···*A*	*D*—H···*A*
N2—H2···O3^i^	0.86	1.97	2.803 (4)	162
N3—H3A···O2^i^	0.86	2.18	3.017 (4)	165
N3—H3A···O2^ii^	0.86	2.43	2.967 (4)	121
N3—H3B···O4	0.86	2.10	2.716 (5)	128
O5—H51···O3^iii^	0.82	1.85	2.670 (4)	180
C4—H4···O1^iv^	0.93	2.42	3.197 (6)	141
C5—H5···O4^v^	0.93	2.30	3.216 (6)	167

Symmetry codes: (i) *y*+1/2, −*x*, *z*−1/4; (ii) −*y*+1/2, *x*, *z*−1/4; (iii) −*y*, −*x*+1/2, *z*−1/4; (iv) *y*, *x*−1/2, *z*−1/4; (v) −*x*+1/2, *y*−1/2, *z*.
